# Decolonization of asymptomatic carriage of multi-drug resistant bacteria by bacteriophages?

**DOI:** 10.3389/fmicb.2023.1266416

**Published:** 2023-11-23

**Authors:** Mehdi Bonnet, Catherine Eckert, Régis Tournebize

**Affiliations:** ^1^Département de Bactériologie, Hôpital Saint-Antoine, AP-HP, Sorbonne Université, Paris, France; ^2^Centre d'Immunologie et des Maladies Infectieuses, INSERM, U1135, Sorbonne Université, Paris, France; ^3^Paris Centre for Microbiome Medicine (PaCeMM) FHU, Paris, France; ^4^Institut Pasteur, Université Paris Cité, Photonic Bio-Imaging, Centre de Ressources et Recherches Technologiques (UTechS-PBI, C2RT), Paris, France

**Keywords:** decolonization, microbiota, bacteriophage, multidrug resistant bacteria, *Enterobacterales*

## Abstract

Antimicrobial resistance is a major threat to human and animal health and accounted for up to 4.5 million deaths worldwide in 2019. Asymptomatic colonization of the digestive tract by multidrug resistant (multi-resistant) bacteria such as extended-spectrum beta-lactamase-, or carbapenemase- producing *Enterobacterales* is (i) a risk factor for infection by these multi-resistant bacteria, (ii) a risk factor of dissemination of these multi-resistant bacteria among patients and in the community, and (iii) allows the exchange of resistance genes between bacteria. Hence, decolonization or reduction of the gastrointestinal tract colonization of these multi-resistant bacteria needs to be urgently explored. Developing new non-antibiotic strategies to limit or eradicate multi-resistant bacteria carriage without globally disrupting the microbiota is considered a priority to fight against antibiotic resistance. Probiotics or Fecal Microbiota Transplantation are alternative strategies to antibiotics that have been considered to decolonize intestinal tract from MDR bacteria but there is currently no evidence demonstrating their efficacy. Lytic bacteriophages are viruses that kill bacteria and therefore could be considered as a promising strategy to combat antibiotic resistance. Successful decolonization by bacteriophages has already been observed clinically. Here, we discuss the current alternative strategies considered to decolonize the digestive tract of multidrug resistant bacteria, briefly describing probiotics and fecal microbiota transplantation approaches, and then detail the *in vivo* and *in vitro* studies using bacteriophages, while discussing their limits regarding the animal models used, the characteristics of phages used and their activity in regards of the gut anatomy.

## Introduction

The unceasing increase of infections by multidrug resistant (MDR) bacteria and their global spread among community and health care settings are a major public health concern. The panel of antibiotics available to treat infections by MDR bacteria can be quickly limited leading to situations of therapeutic « dead end » that are not rare nowadays. Indeed, antimicrobial resistance (AMR) is estimated to have been responsible of between 3.62–6.57 million deaths in 2019 with death rate/100000 ranging from 27.3 in western sub-Saharian countries to 6.3 in Australasia (Antimicrobial Resistance Collaborators, 2022). According to a Review on Antimicrobial Resistance at the British government request, this figure is expected to double to reach 10 million by 2050 (Resistance, 2016). Among the large number of bacteria of medical interest, the ESKAPE group (*Enterococcus faecium*, *Staphylococcus aureus*, *Klebsiella pneumoniae* and *Escherichia coli*, *Acinetobacter baumannii*, *Pseudomonas aeruginosa*, *Enterobacter* sp) represents a group of pathogens for which new therapeutics are urgently needed. Indeed in 2017, the World Health Organization (WHO) listed carbapenem-resistant (CRE) and extended-spectrum beta-lactamase (ESBL) producing *Enterobacteriaceae* (mainly *E. coli* and *K. pneumoniae*), carbapenem-resistant *Pseudomonas aeruginosa* and *Acinetobacter baumannii* as bacteria of critical priority for research and development of new therapeutic strategies (Tacconelli and Magrini, 2017). More than 600,000 deaths are associated to each *E. coli*, *S. aureus*, *K. pneumoniae* and *Streptococcus pneumoniae* infections because of AMR (Antimicrobial Resistance Collaborators, 2022).

Intestinal colonization by MDR bacteria is a risk factor of infections by these bacteria and also represents a reservoir of antimicrobial resistance genes (ARG), with a risk of spread to other patients and within the community and transfer to other bacterial species (Crits-Christoph et al., 2022). Fighting antimicrobial resistance and its consequences is thus a priority. In this context, decolonization strategies aiming to reduce or eradicate gut carriage of MDR bacteria are proposed to be a way to decrease AMR burden. This approach would be useful for patients with a higher risk of infection, such as, for instance, those identified as MDR bacteria carriers in intensive care units or immunocompromised patients. The use of antibiotics to target MDR bacteria is currently the main decolonization strategy that has been developed and reported in studies (Bar-Yoseph et al., 2016). However, 3 systematic reviews conclude that there is a lack of convincing evidence to recommend the implementation of routine digestive decolonization with antibiotics (Bar-Yoseph et al., 2016; Catho and Huttner, 2019; Tacconelli et al., 2019). Moreover, their use is controversial because of the risk of selecting and consequently disseminating resistant mutants. Another consequence of the antibiotics use is the gut microbiota disruption, which is a risk factor for colonization by *Clostridioides difficile* and subsequent infection (Langdon et al., 2016). Therefore, other alternative strategies to decolonize MDR bacteria carriers are needed: among them, fecal microbiota transplantation (FMT), probiotics or the use of bacteriophages are promising and being more investigated. In this review, we aim to summarize the current alternative strategies considered to decolonize the digestive tract of MDR bacteria with a focus on 2 major *Enterobacterales: E. coli* and *K. pneumoniae*.

## Colonization

*Enterobacterales* like *E. coli* or *K. pneumoniae* resistant to third generation cephalosporins (mainly by Extended Spectrum Beta-Lactamases or ESBL production) or carbapenems (for instance by carbapenemase production) are considered as MDR bacteria. The genes encoding for these resistant phenotypes are often carried by mobile genetic elements such as plasmids, that are often carrying multiple antimicrobial resistant genes and that can spread between different species of bacteria. Indeed the important antibiotic usage in the hospital leads to high ESBLs concentration that favors increased conjugation and spread of resistance within the gut microbiota (Sommer et al., 2009; Tschudin-Sutter et al., 2017).

The acquisition of these MDR bacteria and their persistence in the digestive tract are asymptomatic. However this colonization is a source of MDR strains transmission between patients, subsequently responsible for infection or epidemics within healthcare establishments, but also in the community (Vink et al., 2020). The epidemiology of intestinal MDR bacteria carriage has been recently described in few reviews to which the reader can refer to for details (Arzilli et al., 2022; Campos-Madueno et al., 2023). The risk of progression from colonization to infection during hospitalization is estimated at 11% for patients colonized with MDR Gram negative bacteria (Arzilli et al., 2022) and 16.5% for patients colonized with carbapenemase producing *Enterobacteriaceae* (CPE) (Tischendorf et al., 2016). Infections by MDR bacteria have been shown to be associated with high mortality rates, particularly for CPE (Tischendorf et al., 2016; Arzilli et al., 2022). The most frequently reported infections following colonization are pulmonary infections, followed by urinary tract infections, bacteremia and finally skin and soft tissue infections and surgical site infections (Tischendorf et al., 2016; Arzilli et al., 2022).

In hospital setting the rates of MDR bacteria can vary between 12 and 65% (Campos-Madueno et al., 2023). For instance at the time of admission, in a Turkish study, screening of the patients showed that 39.8% of patients were positive for ESBL and 12.5% for carbapenem resistant *E. coli* or *K. pneumoniae* (Kizilates et al., 2021). In France in 2014, 5.3% of the patients were ESBL carriers in an ICU (Jalalzaï et al., 2018). However antibiotic consumption, previous and length of hospital stay, type of ward, patients’ conditions or the geographic location largely impact these data. A systematic review from Arzilli and colleagues showed that the risk of acquiring Gram-negative MDR bacteria during a hospital stay is significant (9.4%). This risk varies significantly according to pathogen and type of hospital units, with notably higher rates for the *Klebsiella* genus (26.4%) and in high-risk units such as intensive care units (10.8%) (Arzilli et al., 2022). Recent hospitalization or treatment with a beta-lactamase inhibitor/beta-lactam combination or carbapenem are also important risk factors for MDR *Enterobacterales* colonization (Detsis et al., 2017).

Since the first description of ESBL-producing bacteria fecal carriage in 2001 (Mirelis et al., 2003), numerous reports have documented the evolution of the fecal prevalence of community carriage rates of MDR bacteria (Woerther et al., 2013). Several meta-analysis indicate a general increase in MDR bacteria carriage, with average carriage rates of around 15% worldwide but with notable regional differences. The figures can be as low as 2% in America, 4% in Europe, and reaching up to 15% in the eastern Mediterranean, 22% in Southeast Asia and Africa, and 46% in West Pacific (Karanika et al., 2016). A recent meta-analysis has estimated the pooled prevalence of ESBL *E. coli* carriage in the community to 16.5% with an 8-fold increase since the beginning of the century (Bezabih et al., 2021). For instance, in a genomic investigation of ESBL-producing *Enterobacterales* strains circulating in Singapore, the rate of ESBL carrier was 26.2% in healthy Singaporeans and 7.4% were carrying a *mcr*1 mutation conferring resistance to colistin, drug used as a last line antibiotic (Ding et al., 2021). In Taiwan, the colonization rate by *E. coli* or *K pneumoniae* resistant to 3^rd^ generation cephalosporin (3GC) or presenting an-ESBL phenotype were 41.4 and 27.4%, respectively, (Huang et al., 2020). A smaller study with 85 participants performed in Mexico among healthy blood donors revealed that 84.7% of the participants were carriers of ESBL-producing bacteria, with ESBL *E. coli* found in 74.1% of the cohort (Tamez-Torres et al., 2020). Data for carbapenem resistant *Enterobacterales* carriage in the community are scarce. A scoping review published in 2017 showed large variability in CRE prevalence rates in various samples, ranging from 0 to 29.5% (Kelly et al., 2017). For instance, a recent study showed that carbapenemase producing *Enterobacterales* carriage rate in Pakistani urban community was 14.4% (Habib et al., 2022). In the community, international travel, particularly to countries endemic for MDR bacteria, appears to be a major risk factor for their acquisition. The rate of acquisition of MDR *Enterobacterales* after international travel varies according to the studies, from 21 to 60% depending on the countries visited. Antibiotic therapy, particularly with beta-lactam antibiotics, as well as the occurrence of gastroenteritis during the stay are shown to be additional risk factors (Hassing et al., 2015; Schwartz and Morris, 2018). In addition, AMR gut carriage following travel has been show to increase the spread of those bacteria within the household [reviewed in Schwartz and Morris (2018)].

## Decolonization

The concept of decolonization, generally referred to in the literature as “decolonization,” “loss of carriage” or “eradication” refers to the eradication of carriage of MDR strains. Yet, to date, there is no agreed definition of digestive decolonization of any MDR bacteria. In particular, it has been shown that the number of negative rectal swabs chosen to affirm loss of carriage influences the decolonization rate found in different studies. Although not statistically significant, higher decolonization rates were reported when a single negative sample is used to define decolonization compared with studies that require more than one. Higher rates of decolonization are also reported in studies in which resistant strains are identified only by phenotypic method, compared with those in which strains are also identified by genotypic method (Bar-Yoseph et al., 2016). It is also important to note that it is very difficult to routinely distinguish persistent carriage from reacquisition. While the main reservoir of Gram negative bacteria in humans is the digestive tract, several other locations have already been described in the literature as sites of carriage, notably the urinary tract and inguinal folds (Weintrob et al., 2010). These other sites could potentially be sources of reacquisition of the MDR strain in the same patient, perhaps highlighting the value of using decolonization strategies that are also active extra-intestinally.

One has to keep in mind that colonization by MDR bacteria can be dynamic and fluctuate in time. In the course of the natural history of digestive MDR bacteria carriage, spontaneous decolonization without intervention can occur. This is highlighted in a recent meta-analysis where 50% of ESBL carriers spontaneously decolonize in 2 months, while about 20% remain colonized up to 18 months after initial colonization. This occurs in patients discharged from hospitals, travelers or in the community (Ling et al., 2022). In a literature review by Bar-Yoseph et al. the proportion of patients colonized with ESBL -producing *Enterobacterales* in healthcare facilities declines over time, but still persists at 1 year (around 80% at 1-month follow-up, 56% at 6 months and 36% at 1 year) (Bar-Yoseph et al., 2016). These observations show that still a significant proportion of the population is unable to clear its microbiota from ESBL-producing bacteria, and hence remain a vector for AMR transmission and colonization within the community. A study by Riccio et al. has actually observed a household ESBL acquisition rate of ESBL from patient discharged from the hospital. This rate is 41% upon one week returning one housing, and 29% after two months. In this study, only one third of the index cases turned ESBL-negative after 3 months (Riccio et al., 2021). Spontaneous natural decolonization of ESBL-producing bacteria is thus not an option. In the hospital setting, colonization by MDR bacteria is a risk factor for developing infections or cross-transmission to other patients and hygiene and stewardships measures have proved to be effective in preventing cross-transmission.

Among the strategies considered to achieve digestive decolonization of MDR bacteria, the use of antibiotics accounts for the vast majority of data available to date. Given the limitations of this approach, other alternative strategies have been discussed and studied: the use of probiotics, FMT and bacteriophages.

## Antibiotherapy

The vast majority of published data on digestive decolonization of MDR bacteria concerns the use of antibiotics to eradicate carriage of MDR *Enterobacterales*. Colistin and aminoglycosides (gentamicin, streptomycin, neomycin in particular) are the most widely used antibiotics for this purpose, alone or in combination, since they are not absorbed when administered orally and have no activity against the anaerobic bacteria of the microbiota. Out of 13 included studies on the decolonization of ESBL-producing *Enterobacterales* by antibiotics (mainly aminoglycosides and/or colistin), the rate of colonized patients is 37% at the end of antibiotic therapy for decolonization, and rises to 58% 1 month later (Bar-Yoseph et al., 2016). Similar results are observed in a randomized controlled trial of 58 patients showing a significantly lower rate of rectal carriage at the end of treatment in those receiving antibiotic therapy (colistin or neomycin for 10 days) aimed at decolonization compared with a group receiving placebo (32% vs. 77%), with however a disappearance of the effect at day 7 and day 28 post-treatment (Huttner et al., 2013).

However, the use of antibiotics for decolonization exposes patients to the risk of emerging resistance to these antibiotics. This is particularly important for colistin, which is a treatment of last resort for MDR Gram-negative bacterial infections (Yang et al., 2020). The few randomized trials that have looked at this as a secondary endpoint show no increase in colistin resistance in the treated group (Saidel-Odes et al., 2012; Huttner et al., 2013; Stoma et al., 2018). However, a prospective controlled trial from 2013 showed the emergence of secondary resistance to the antibiotics used for decolonization in 14% of patients treated, with notably 1 case of colistin resistance in 16 patients and 6 cases of gentamicin resistance in 26 patients (Oren et al., 2013). Hence, the current literature of clinical trials does not provide sufficient data to assess the emergence of resistance to antibiotics used for the eradication of digestive carriage of MDR bacteria. In addition, a growing body of literature demonstrates the emergence of colistin resistance, for instance, in *Enterobacterales* such as *E. coli* and *K. pneumoniae* (Bastidas-Caldes et al., 2022; Uzairue et al., 2022). It therefore seems crucial to carefully take into account this phenomenon before considering wider clinical application.

Unlike to what has been described for nasal decolonization of methicillin resistant *S. aureus* (Sharara et al., 2021), antibiotherapy has not shown a clear decolonization efficacy of the digestive tract (Tacconelli et al., 2019). To date, the review by Taconelli et al. constitutes the data reference on which the European recommendations of the European Society of Clinical Microbiology and Infection Diseases - European Committee on Infection Control (ESCMID-EUCIC) are based and do not recommend routine decolonization of ESBL and CRE bacteria carriage (Tacconelli et al., 2019). However, antibiotic decolonization of MDR *Enterobacterales* may be considered in specific patient populations, such as neutropenic patients, but with limited evidence (Tacconelli et al., 2019). Randomized controlled trials are needed to assess the potential efficacy of antibiotic decolonization, specifically targeting high-risk patients, such as those with hematological pathologies or solid organ transplants colonized during hospitalization.

## Probiotics

An alternative strategy to antibiotics for the digestive decolonization of MDR bacteria is the use of probiotics that are defined by the WHO as living micro-organisms that confer a health benefit on the host when administered in adequate quantities. Probiotics are already widely used for example in prevention of antibiotic associated diarrhea. *Lactobacillus* sp., *Bifidobacterium* sp., *Saccharomyces boulardii* are some examples of the large variety of bacteria that can be used. There is growing public and scientific interest in these micro-organisms with the aim of preventing or treating pathologies (Seale and Millar, 2013). However analysis of 14 clinical studies failed to show a benefit of probiotics in decolonization of MDR bacteria or in prevention of acquisition of MDR bacteria, supporting the ESCMID guidelines (Tacconelli et al., 2019; Karbalaei and Keikha, 2022). Hence, there is currently no evidence demonstrating the efficacy of probiotics on the decolonization of MDR bacteria, and consideration must be given to the possible impact on the gut microbiota and its consequences, as well as the risks of infection associated with these microorganisms, particularly in immunocompromised patients (Didari et al., 2014). However, several parameters such as the type of probiotic strains used, dosage, frequency of administration could be more precisely defined in order to try to improve probiotics utilization for MDR bacteria decolonization. Indeed, few clinical and fundamental observations suggest that probiotics could be useful. For instance in a preliminary study conducted in Italy, 36 patients colonized by a carbapenemase producing *K. pneumoniae*, received either usual care or usual care + probiotic treatment for 14 days (Nouvenne et al., 2015). Loss of the CRE *K. pneumoniae* of stool samples was observed in 10 patients receiving probiotics versus 2 patients in the control group. Although these results seems promising, no data are available regarding the composition of the commercial symbiotic treatment used, nor about the kinetic of decolonization of the samples. In a more recent fundamental study, *Klebsiella oxytoca* was shown to outcompete carbapenemase producing *K. pneumoniae* strains in a murine model (Osbelt et al., 2021). In this work, mice were co-colonized or pre-colonized with *K. oxytoca* before colonization with a carbapenem resistant *K. pneumoniae* strain. Elimination of carbapenem resistant *K. pneumoniae* was successfully completed in 80% of the mice after 42 days, versus 20% of the control group. To confirm these results in a more complex model, the authors used humanized microbiota mice that had received microbiota from either children or adults human donor. In these mice, *K. oxytoca* can impair MDR *K. pneumoniae* outgrowth by competiting for carbon sources, with different clearance kinetics depending of the human microbiota used. Overall, despite current absence of recommendation for the use of probiotics for MDR bacteria decolonization, this approach should still be considered as current research on the microbiota provides a wealth of new information about the role, function and impact of some commensals bacteria and their potential use as probiotics, as seen for instance with the development of *Faecalibacterium prausnitii* as a new generation probiotic for inflammatory bowel diseases (Martín et al., 2023).

## Fecal microbiota transplantation

The gut represent a huge reservoir of antimicrobial resistant genes (Transferable drug resistance, 1969). So far, modify or replace the intestinal microbiota can represent an interesting strategy to decolonize the digestive tract from MDR bacteria. Fecal microbiota transplantation (FMT) consists of transferring the fecal microbiota of a healthy donor, from a stool sample, to the patient’s gastrointestinal tract. This therapeutic strategy is notably recognized as safe and effective for the treatment of patients with recurrent *C. difficile* infections and is now the subject of international recommendations in this context (Debast et al., 2014; McDonald et al., 2018). FMT can also be considered as a strategy for eradicating digestive carriage of MDR bacteria. A 2019 systematic review conducted by Yoon et al. mentions the potential benefit of using FMT in the setting of digestive decolonization of MDR bacteria, but most of the studies included are not randomized controlled trials and thus sufficient evidence is still lacking to confirm this hypothesis (Yoon et al., 2019). A more recent systematic review, including 7 studies and 5 cases reports came to similar conclusions (Bilsen et al., 2022). Decolonization rates after FMT varied from 20 to 90% but the studies were heterogenous (pre- or post-treatment with antibiotic or not, type of FMT procedure, types of MDR bacteria, number of patients included, duration of follow-up). For instance one of the largest study including 35 patients showed promising results with 68.5% of decolonization rate at one year (Seong et al., 2020). In this study only 4 patients were colonized with carbapenemase strain (mostly *K. pneumoniae* carbapenemase producing strain) and 12 were colonized with both carbapenemase strain and vancomycin resistant *Enterococcus*. Among the 4 patients colonized with carbapenemase producing strain, 3 were successfully decolonized. A first multi-centric randomized clinical trial including 39 patients failed to obtain statistically significant results due to early termination of the study despite a slightly decreased of ESBL and/or carbapenemase producing *Enterobacterales* colonization compared to the control group: 41% of the patients colonized were negative at day 35–48 compared to 29% of the patients of the control group (Huttner et al., 2019). A second randomized double blind trial study against placebo (KAPEDIS) regarding the efficacy of decolonization of KPC producing *K. pneumoniae* is currently ongoing (Perez-Nadales et al., 2022). So far, according to Merrick et al. 13 randomized studies are actually ongoing or have been recently completed (Merrick et al., 2023). Number of participants recruited, inclusion criteria and primary and secondary outcomes of those studies show significant differences between the studies. 7/13 of those studies are randomized, double-blind, controlled trials; 5/13 are randomized, open-label, controlled trials, and 1/13 trial is randomized, participant- blinded, controlled. The number of estimated included participants ranges between 9 and 437 with 6/13 studies including more than 100 participants. The recruited participants have different origin, including adults inpatients colonized with MDR bacteria, to specific population having had renal transplantation. Colonizing bacteria are mainly ESBL-producing *Enterobacterales* or CRE identified either by culture or PCR. The fecal material is administrated mainly by capsule (7/13), retention enema (4/13), colonoscopy (1/13), and nosoduodenal tube (1/13). The primary outcome is decolonization (evaluated by culture or PCR) but at varying times post treatment with assessment ranging from 3 days to 6 months, with 9/13 studies with an evaluation set at 1 month. Overall, despite their difference in the studies design, those ongoing randomized clinical trial highlight the growing interest of this low risk therapeutic to decolonize MDR bacteria. Their results, when published, will hopefully provide a clearer view of FMT decolonization efficacy and potential future utilization.

## Bacteriophages

Bacteriophages (or phages) are bacterial viruses discovered over 100 years ago by Frederick Twort and Félix d’Hérelle, who observed the presence of lysis halos on bacterial cultures (Twort, 1915; D'Herelle, 1917). D’Hérelle was the first to call these viruses “bacteriophages,” literally “bacteria eaters” and was also the first to successfully conduct phage therapy to treat typhoid in chickens: this was the birth of phagotherapy. The development of phage therapy took place mainly in the first half of the 20th century, before the era of antibiotics, and phages were used to treat a wide range of infections, with significant therapeutic success, particularly in the absence of alternatives at the time (Marongiu et al., 2022; Stacey et al., 2022).

Bacteriophages are ubiquitous and found in all ecosystems; they represent the largest biomass on Earth with an estimated number of 10^32^, for 10^8^ different genomes (Rohwer, 2003; Chibani-Chennoufi et al., 2004) and over 6,000 species listed by the International Committee on Taxonomy of Virus (Krupovic et al., 2021). They can carry different type of genetic materials (ssRNA, dsRNA, ssDNA, dsDNA), and have various structures and shapes.

There are two types of bacteriophage cycle: the “lytic” (or virulent/productive) cycle and the “lysogenic” (or temperate/dormant) cycle. The lysogenic cycle involves the integration of the phage’s genetic material into the chromosome of the infected bacterium, forming a dormant prophage, without destruction of the bacterial cell or production of new bacteriophages. The phage genetic material is then transmitted vertically to daughter cells and also sometimes by horizontal transfer to neighboring bacteria. Prophages can be re-activated under the influence of abnormal environmental conditions or other external stress, restart replication and lytic infection cycle. The lytic cycle takes place in several phases, chronologically involving adsorption, injection of the genetic material contained in the capsid, replication, assembly of new viral particles and then lysis of the infected bacteria, enabling the release of new virions. Bacteriophages characterized by a lytic cycle are those of clinical interest, since they induce a rapid and highly specific bactericidal effect. For this reason, they are seen as an alternative to tackle multidrug resistance in bacteria.

Numerous parameters and characteristics specific to each bacteriophage-host bacterium pair need to be characterized *in vitro* to guarantee successful use for clinical purposes, in particular burst size and multiplicity of infection (MOI). The burst size corresponds to the number of phages released by a lysed bacterium, while the MOI corresponds to the bacteriophage/bacterium ratio and is used to define the initial phage inoculum to be administered in *in vitro* and *in vivo* studies (Glonti and Pirnay, 2022).

Bacteriophages offer a number of advantages for their use as therapeutic agents, particularly in comparison with antibiotics. Firstly, phages are highly specific for their target bacteria, enabling them to be active only on the pathogen to be eliminated while preserving the microbiota: their host spectrum is very narrow (Wittebole et al., 2014). Moreover, their replication at the site of infection can take place from the very first application and, unlike antibiotics, allows their concentration at the site of infection to increase over time. Moreover, several clinical studies on efficacy and/or safety of phagotherapy indicate that this approach is safe. Side effects that have been reported are generally accounted for by inflammatory bacterial contamination (Stacey et al., 2022). Moreover, reassessment of the old clinical trials that have been performed indicate that phagotherapy is efficient (Marongiu et al., 2022), even though, efficiency of some recent clinical trials has been observed only in 2 out 7 clinical trials. Therapeutic efficiency requires the bacteriophages to be delivered to the right infectious sites and that the infectious sites harbors enough bacteriophage-sensitive bacteria (Stacey et al., 2022). Overall, the clinical trials that have been performed have allowed to better define the conditions more suited for an efficient therapy that will permit in the future to better assess phagotherapy efficacy.

It should be noted that legislation concerning bacteriophages does not facilitate their use for clinical purposes: health authorities of western countries generally supervises and authorizes their availability and use only within the framework of clinical trials or in a compassionate setting, notably for cases of infections by MDR bacteria escaping all lines of antibiotic therapy.

There are, of course, limits to the clinical use of bacteriophages. Their highly specific nature with regard to their host bacteria means that they have a very narrow spectrum of activity, sometimes limited only to particular strains or clones. In addition, bacteriophages can degrade rapidly under certain environmental conditions, and their stability and long term storage condition should be tested and frequently checked before being used in humans. Furthermore, only bacteriophages with a lytic cycle can be used for clinical purposes, while those with a lysogenic cycle may be responsible for horizontal transfer of antibiotic resistance genes (Górski et al., 2009). Finally, bacterial resistance to bacteriophages is also a well-documented mechanism that could hinder the utilization of bacteriophages. Bacteria possess a large panel of defence mechanisms ranging from mutating the bacteriophage receptor (LPS, capsule, outer membrane proteins, other transporters…), degrading the viral genetic material to expressing CRISPR_Cas or restriction-modification systems for instance that negatively affect the infectivity of the bacteriophages (Roach and Debarbieux, 2017; Egido et al., 2022). One approach to limit the emergence of such clones is to use a cocktail of different bacteriophages that are using different receptors for entry into bacteria. As emergence of a resistant clone is the selection of a stochastic mutant within a large population, the likelihood to select for bacteria that are mutated in several receptors is extremely low. Moreover, in the context of infection, the selection of bacteriophage-resistant bacteria can be detrimental to the bacteria as mutations of several types of phage receptors such as capsule, LPS or other receptor can lead to reduced bacterial virulence and increased sensitivity to the host immune system leading to clearance of the pathogen from the organism (Egido et al., 2022).

Use of phage as therapeutics have proven efficacy in some case reports and in compassional use but data for decolonization are lacking and need more studies to be implemented in clinic. In the following section, we detail studies addressing decolonization of MDR bacteria by bacteriophages in *in vitro*, animal or human models, with an emphasis on *E. coli* and *K. pneumoniae* as models of the main MDR *Enterobacterales*. Studies concerning the treatment of gastrointestinal infections have already been discussed elsewhere (Javaudin et al., 2021b) and will not be considered here, as most studies involving non-MDR bacteria that are beyond of the scope of this review focusing on decolonization of MDR strains.

### *In vitro* models

#### Escherichia coli

Recapitulating partly the gut environment *in vitro* allows to quickly test and evaluate decolonization strategies. Bernasconi et al. used a simplified *in vitro* model of digestive tract colonization consisting of a fermenter inoculated with two pools of stools from healthy donors free of ESBL- or carbapenemase- producing *Enterobacterales* (Bernasconi et al., 2020). Colonization was achieved after inoculating these stools with a CTX-M-15 ESBL-producing strain of *E. coli*. After 3 consecutive introduction of a sterile filtrate of bacteriophages lysate (10^5^ to 10^6^ PFU/mL) of several pathogenic *E. coli, Shigella* spp., *Salmonella* spp.*, Proteus vulgaris/mirabilis, Pseudomonas aeruginosa, Staphylococcus* spp. *and Enterococcus* spp., a significant decrease of the MDR strain population and the absence of restoration of this population after cessation of treatment were observed for one of the two stool pools during the 48-h observation period of the experiment. The specificity of the bacteriophages toward the MDR *E. coli* was observed as the introduction of those phages had no effect on the total *E. coli* population of the microbiota while acting on the specific MDR strain. However, in one of the fermenter batch, the inhibition of the MDR strain was only temporal and the strain regrew. Resistant mutants were isolated and found to have incorporated 2 additional plasmids and presented few Single Nucleotides Polymorphisms. One of them, in a glycosyl transferase family 2 protein domain is hypothesized to be responsible of the bacterial resistance to the phage by increasing the sugar content on the membrane thereby potentially blocking one or more phage receptors. A major limitation of this study is that it approximates the environment represented by the human digestive tract, thus neglecting possible interactions between the host’s digestive tract cells, its microbiota, the bacterium of interest and the bacteriophages. Moreover, this *in vitro* model was built under aerobic conditions, preventing the growth of strictly anaerobic bacteria physiologically present in the human digestive tract.

Another *in vitro* work tried to assess the combined benefit of using phage to reduce the bacterial load of an MDR bacteria with the competitive action of a probiotic preventing the outgrowth of phage-resistant MDR bacteria (Laird et al., 2022). A bacteriophage specific of a CTX-M-1 ESBL-producing MDR *E. coli* was used in combination with a probiotic corresponding to a commensal wild type *E. coli* strain producing colicin bacteriocin. On the one hand, the bacteriophage alone incubated with the ESBL *E. coli* strain lead to a sharp decrease in the number of bacteria, followed by regrowth due to the selection of a bacterial mutant. On the other hand, when the commensal and the ESBL strains were co-cultured together, the growth of the ESBL strain was transiently inhibited by up to 3 log depending on the commensal strain used, indicating that the commensal strain can reduce, but not abolish the growth of the ESBL bacteria by exerting a competitive exclusion mechanism. However, when the bacteriophage and the commensal were used in combination, the bacteriophage quickly killed the majority of the ESBL-producing bacteria, and the commensal outcompeted the ESBL-producing strain, completely abolishing its regrowth. Hence, *in vitro*, a combined action of a bacteriophage and a probiotic was shown to be efficient at decolonizing ESBL-producing bacteria. These experiments need to be confirmed in more complex *in vitro* system mimicking the gut and evaluated in animal models.

### *In vivo* animal models

#### Escherichia coli

A murine model where mice first received an oral treatment with streptomycin and then were colonized with a MDR *E. coli*, was used to assess the efficacy of a cocktail of bacteriophages in decolonizing MDR *Enterobacterales* colonizing the digestive tract, and its impact on microbiota diversity (Galtier et al., 2016). While the mice were under constant antibiotic pressure, when the three bacteriophages were administered individually or in cocktail, only a transient decrease was observed. But when the antibiotic pressure was released after colonization, and upon addition of a cocktail of the three phages, a strong reduction in the bacterial load was observed a week after phage injection, indicating a positive action of the bacteriophage cocktail. Furthemore, by assessing the abundance of different bacterial genera present in the stools through 16S rRNA sequencing, the authors were able to show a significantly lower impact of bacteriophages on the digestive microbiota of mice as compared to antibiotics usage.

The study by Javaudin and colleagues presents less optimistic data than the above-mentioned studies. In their murine model, they were unable to demonstrate the long-term efficacy of using a cocktail of bacteriophages specific to an ESBL *E. coli* (type not specified in the article) producing carbapenemase OXA-48, for digestive decolonization (Javaudin et al., 2021a). They were only able to demonstrate a transient 1-log decrease in the fecal concentration of the MDR strain following oral, oral and rectal administration or microencapsulation of bacteriophages. Similarly, another study showed only a 0.5-log transient decrease in the carriage of a CTX-M-15 ESBL-producing MDR *E. coli* in a mouse colonization model (Porter et al., 2022). However, they observed a significant 3-log decrease in the strain of interest in the stools of the mice studied when the bacteriophage cocktail was combined with a probiotic (Microcine-C7 bacteriocin-producing *E. coli*), suggesting a potential synergy. It should be noted, however, that the effect remains transient (1 week maximum) and declines despite continued treatment.

#### Klebsiella pneumoniae

In a first study by Fang et al. one first phage (P24) targeting a *K. pneumoniae* strain producing carbapenemase type KPC-2 and a phage targeting a capsule-deficient mutant of that strain were isolated, characterized and tested for their capacity to modify the gut colonization by this strain (Fang et al., 2022). Several bacterial mutants resistant to the P24 phage were isolated and shown to be mutated for capsule production. In a murine colonization model, a moderate reduction in the MDR *K. pneumoniae* fecal population (2 log) was observed following rectal administration of a single bacteriophage, but resistant mutants emerged in all mice in the week time-frame of the experiment. When a cocktail of 2 bacteriophages was administered, a greater reduction in MDR *K. pneumoniae* was observed (from 4 to 6 log); the emergence of mutants resistant to both bacteriophages was nevertheless observed in one mouse. Some of the strains resistant to the phages had altered production of capsule or mutation in a cyclic di- GMP phosphodiesterase. These mutants were also shown to be less virulent in a *Galleria mellonella* larvae model.

Two other studies investigated decolonization by bacteriophages of germ-free mice colonized with human microbiota containing a carbapenem-resistant *K. pneumoniae*. A first study from Taiwan used colonized mice with human stool carrying a K64 *K. pneumoniae* producing carbapenemase of the K64 serotype, the main MDR clone circulating in Taiwan (Liu et al., 2022). Here also, two different phages were used alone or in combination. When administered orally, one phage was unable to decolonize the microbiota of the K64 CRE. The other was able to decolonize mice for 2 weeks before recolonization was observed. When the 2 phages were used in combination, most of the mice were also decolonized for 2 weeks. A stronger decolonization for 2 months was observed in half of the animal when the phages were administrated both intragastrically and intraperitonally.

A recent study identified phages targeting either Wild Type or capsule mutants of *K. pneumoniae* (Lourenço et al., 2023). The authors showed that phages targeting capsule mutants had a broader host range and provide increased efficiency at killing *K. pneumoniae* when used in combination with capsule-targeting bacteriophage. When used *in vivo* in OM12 mice, that have a defined model microbiota of 12 species, and colonized with a hypervirulent K2 *K. pneumoniae*, only a temporary 10-100x decrease in the bacteria load was observed when both phages were used alone or in combination. *K. pneumoniae* is known to strongly express a capsule, but the fact that the capsule-independent phage showed some activity indicate that within the gut microbiota, the bacteria is able to modulate its capsule expression. Although the *K. pneunomiae* was not a MDR strain, this study is interesting for phagotherapy approaches, as being able to target with phages both capsulated and acapsulated bacteria could increase potential therapeutic approaches based on phages.

### *In vivo* human model

In 2020, Corbellino et al. reported a clinical case of decolonization with a cocktail of bacteriophages in a patient colonized with carbapenemase-producing KPC-3 *K. pneumoniae* (Corbellino et al., 2020). The patient had a history of Crohn’s disease in remission, and had a complex clinical history with a right nephrectomy and total cystectomy with left ureterostomy and ureteral stenting following recurrent episodes of lithiasis and urinary tract infection complicated by extensive cystic fibrosis. In addition, she presented with multi-site colonization (gastrointestinal tract, urinary tract and on a permanent external invasive device) by a MDR strain of *K. pneumoniae*, resistant to all beta-lactams (with the exception of Ceftazidime-Avibactam). Several episodes of urinary tract infection and sepsis have been documented for this patient with this strain of *K. pneumoniae*, isolated in culture from urine, ureteral stent and blood cultures, and successfully treated empirically with Ceftazidime-Avibactam. However, after each infectious episode, the MDR strain reappeared and persisted in culture at all the sites tested. A cocktail of MDR strain-specific lytic bacteriophages was therefore used to decolonize the patient, administered orally and rectally for 3 weeks. No adverse effects were reported, and the treatment was well tolerated. At the end of the bacteriophage treatment and until publication of the study, the MDR strain was never again isolated in culture on selective media from the sites previously colonized, i.e., from stools, rectal swabs, urine and the ureteral stent. In addition, molecular biology tests for carbapenemase genes on rectal swabs, carried out 5 days after the start of treatment with the phage cocktail and every 7 to 14 days during patient follow-up, were always negative. This is all the more encouraging given the technique’s greater sensitivity than culture on selective media. Interestingly, only 2 other non MDR *K. pneumoniae* have since been isolated from the patient’s stool or rectal swab, but these were sensitive to most antibiotics and belonged to two other clones. This confirms the close specificity of bacteriophages to their host bacteria, and therefore the possibility of highly selective decolonization directed almost exclusively against the strain of interest.

## Conclusion

Decolonization of the gut microbiota from MDR bacteria has not yet been reproducibly achieved. Antibiotics have the drawback of not being selective enough. Bacteriophages however have the advantage of being selective and not alter the microbiota. But they have not yet consistently shown to be effective. We believe that a reason for this is our yet limited knowledge of the interactions between phages and bacteria in the gut.

A parameter that has been overlooked when considering the use of bacteriophages for decolonization is the gut geography and differential localization of bacteria and phages in the digestive tract. The gut epithelium is indeed separated from the luminal microbiota by a mucus layer that protects it from bacteria and bacteria components. However, this barrier is not fully impenetrable and bacteria such as *Acinetobacter*, *Delftia* and *Stenotrophomonas* have been shown to establish niches in the colon crypts of mouse and humans (Pedron et al., 2012; Saffarian et al., 2019). Other bacteria such as *Akkermensia muciniphila*, *Bacteroides* spp. or *Bifidobacterium* spp., but also *K. pneumoniae* (Glover et al., 2022; Hudson et al., 2022), are known to be able to reside in the mucus layer and use it as a carbon source. Also, in mice gut colonization experiments with *E. coli* or *K. pneumoniae*, even though the bacteria are mainly observed in the lumen, some bacteria can be detected in the mucus (Caballero et al., 2015; Lourenço et al., 2020) where they are not accessible to bacteriophages present in the lumen. Similarly, several phages have been shown to interact with mucin (Barr et al., 2013; Almeida et al., 2019; Green et al., 2021; Chin et al., 2022) and the presence of phages in the mucus proposed to provide a non-host immunity against invasion or colonization of the gut mucosa by bacteria (Barr et al., 2013), [reviewed extensively in Rothschild-Rodriguez et al., 2022]. This means that bacteria localized in the mucus are, in the absence of mucus associated-bacteriophages targeting them, not visible from bacteriophages that cannot interact with and penetrate in the mucus and are luminal. These bacteria are thus a constant source of bacterium that, by being shed, can recolonize the lumen (Lourenço et al., 2020), and thus represent an inaccessible hidden reservoir of the bacteria to eradicate. However, they are target of phages that can interact with mucin, and be activated by mucin. Green and colleagues, using a *E. coli* non-MDR ExPEC strain were convincingly able to eradicate the colonizing bacterium with a mucin-targeting bacteriophage (Green et al., 2021). When considering decolonization one should thus consider these different environments and isolate bacteriophages that also have a tropism for mucin. A cocktail of phages that can act in these different compartments might be more efficient at decolonizing the gut from a MDR strain. This capacity to interact with mucin is a parameter that has rarely been considered or discussed so far in the publications addressing gut decolonization by bacteriophages and should be more taken into account. Also, whenever an application of bacteriophages in *in vivo* models or in humans is foreseen, applying a set of standardized protocols to characterize thoroughly the bacteriophages will be useful to anticipate or predict the efficiency of the bacteriophages in clinical applications (Glonti and Pirnay, 2022).

Emergence of phage resistance is a common phenomenon and can be a limitation to the use of bacteriophage. In addition to the classic mutations in the bacterial phage receptors, several new mechanisms of the bacterial defense systems underlying it have been uncovered in the recent years (Bernheim and Sorek, 2020; Tal and Sorek, 2022). The acquisition of phage resistance can come with a fitness cost for the bacterium, which may in return lead to a reduced virulence and increased sensitivity to the host immune response (Fang et al., 2022) or decreased antibiotic resistance profile with the recovery of susceptibility to certain antibiotics (Majkowska-Skrobek, 2021). In order to better understand and predict better efficacy the mechanisms of phage resistance should therefore be investigated both in *in vitro* studies and in the host context (Gaborieau and Debarbieux, 2023). To counter this emergence of resistant mutants, two approaches are envisaged. One relies on the use of cocktails of phages targeting different bacterial phage receptors, in order to minimize phage-resistance evolution. The second approach makes use of bacteriophages that select a bacterial resistance mechanism inducing a reduced virulence or increased sensitivity to antibiotics (Torres-Barceló et al., 2022).

It is still possible that, depending on the context (host, bacteria, bacteriophages), bacteriophages alone will not be sufficient to efficiently eradicate the MDR colonizing bacteria. Complementing MDR bacteria decolonization by phages with the action of a probiotic preventing its outgrowth as observed by Laird and colleagues *in vitro* (Laird et al., 2022) could represent an interesting alternative approach that would need to be further investigated.

## Author contributions

MB: Conceptualization, Writing – original draft. CE: Conceptualization, Supervision, Writing – review & editing. RT: Conceptualization, Supervision, Writing – review & editing.
